# Diversity in mental fatigue and social profile of patients with myasthenia gravis in two different Northern European countries

**DOI:** 10.1002/brb3.653

**Published:** 2017-03-01

**Authors:** Liis Sabre, Elisabet Westerberg, Maarika Liik, Anna R. Punga

**Affiliations:** ^1^ Department of Neuroscience, Clinical NeurophysiologyUppsala University and Uppsala University HospitalUppsalaSweden; ^2^ Department of NeurologyTartu University HospitalTartuEstonia

**Keywords:** Estonia, fatigue, myasthenia gravis, Sweden

## Abstract

Self‐estimated health can be used for comparison of different diseases between countries. It is important to elaborate on whether disparities in self‐estimated health are due to disease‐specific parameters or socioeconomic differences. In this study, we aimed at evaluating clinical and social similarities and differences in myasthenia gravis (MG) patients between comparable regions in two Baltic Sea countries, Estonia and Sweden.

**Methods:**

This cross‐sectional study included southern counties in Sweden and Estonia of comparable size. All patients with a confirmed MG diagnosis were asked to answer two questionnaires including demographic and disease‐specific data, lifestyle issues, and mental fatigue (Fatigue Severity Scale [FSS]). Clinical fatigue was assessed objectively through the Quantitative Myasthenia Gravis Score (QMG).

**Results:**

Thirty‐six of 92 identified patients in Estonia and 40 of 70 identified MG patients in Sweden chose to participate in the study. The demographic characteristics and symptoms reported by the patients were similar. QMG score did not differ; however, the Estonian patients scored their current subjective disease severity significantly higher (5.6 ± 2.8) compared to the Swedish patients (3.4 ± 2.3, *p* = .0005). Estonian patients also had significantly higher FSS scores (5.0 ± 1.7) than Swedish patients (3.5 ± 1.6; *p* = .001). Swedish patients were more active and performed physical activity more regularly (29.1% in Estonia and 74.2% in Sweden, *p* = .004).

**Conclusions:**

Although, the patients had comparable clinical fatigue, Estonian patients evaluated their health state as being more severe and reported more mental fatigue than Swedish patients. These data indicate large regional differences in disease perception of MG, which is important to consider in international studies.

## 1 Introduction

Although myasthenia gravis (MG) is a rare disease, the majority of epidemiological studies on MG are limited to national areas, whereas one previous study has compared regions from two different European countries, Norway and the Netherlands (Boldingh et al., 2015). The prevalence of disease‐specific antibodies is known to vary in different regions of the world, in particular the prevalence of muscle‐specific tyrosine kinase (MuSK) antibodies (Gilhus & Verschuuren, 2015). Nevertheless, more subtle differences such as disease perception and the influence of social factors may be more difficult to pinpoint. The annual incidence of MG is around 10 cases per million and the prevalence about 150 per million (Carr, Cardwell, McCarron, & McConville, 2010; Gilhus, Nacu, Andersen, & Owe, 2015). Therefore, multicenter studies are essential in research of rare diseases in order to fill the knowledge gaps in pathogenesis, geographical differences, natural history, and treatment of the diseases. In such studies, it is important to consider both disease‐related as well as more general differences between different patients and countries.

To pinpoint some of these issues, we decided to perform a comparative study of MG in two European regions: Jönköping county in southern Sweden and the counties of Southern‐Estonia. These regions are similar in the number of inhabitants, north‐south gradient, and climate, and the quality of health care is not different according to the World Health Organization (Lai et al., 2013). However, lower socioeconomic level in Estonia has been described, for example, in two previous comparative studies between Scandinavian and Estonian patients with neurological diseases (Rekand et al., 2003, 2004; Sabre, Hagen, Rekand, Asser, & Korv, 2013). Therefore, we decided to use uniform inclusion criteria, case identification system, homogenized clinical scales, and questionnaires to find clinical, social, and psychological similarities and differences in MG patients in these comparable regions.

## 2 Methods

### 2.1 Study design

The patient cohorts originated from two different epidemiological studies in equally sized regions of Estonia and Sweden. Valga, Võru, Viljandi, Põlva, Tartu, and Jõgeva counties in Estonia and Jönköping county in Sweden were included in the study. The Estonian study was approved by the Research Ethics Committee of the University of Tartu, Estonia (245/T‐12) and the Swedish study was approved by the Research Ethics Committee in Linköping University, Sweden (Dnr 2014/459‐31). All participants gave their written informed consent.

According to the census on 1 January 2015, there were 319,782 inhabitants living in the six counties of Estonia and 344,262 inhabitants in Jönköping county of Sweden on 31 December 2014. In Estonia, Tartu University Hospital served as the regional hospital in the six counties, and in Sweden, Jönköping county was served by regional hospitals in Eksjö, Jönköping, and Värnamo. Medical records from Tartu University Hospital and Eksjö, Värnamo, and Jönköping hospitals were collected.

### 2.2 Patients and inclusion criteria

#### 2.2.1 Inclusion criteria

According to the Myasthenia Gravis Foundation of America (Jaretzki et al., 2000), MG diagnosis was confirmed if ≥2 of the following criteria were fulfilled: (1) objective clinical fatigue based on neurologist consultation; (2) disturbed neuromuscular transmission on repetitive nerve stimulation (RNS) or single‐fiber electromyography; and (3) presence of serum antibodies against acetylcholine receptors (AChR+) or muscle‐specific tyrosine kinase (MuSK+).

#### 2.2.2 Estonian cohort study

Medical data of the patients with the diagnostic code G70.0 or G70.9 according to ICD‐10, suggesting MG, were reviewed at Tartu University Hospital. In the Estonian study, 104 patients with MG diagnosis according to ICD code were identified from 2005 to 2014 from the medical records. Among these, 12 were dead and medical records of 92 cases met the inclusion criteria of MG (Jaretzki et al., 2000). Fifty‐three individuals agreed to take part in the study (response rate 58.2%); however, merely 36 Estonian patients remained in the study after objective examinations by the researchers (ocular symptoms in the beginning that rapidly disappeared, symptoms not characteristic of MG, lack of neuromuscular transmission failure, seronegativity for AChR and MuSK antibodies). There was no significant difference between responders and nonresponders (14 men vs. 39 women among responders, mean age 59.4 ± 16.9 years; 16 men vs. 23 women, mean age 65.9 ± 18.3 years, *p* > .05).

#### 2.2.3 Swedish cohort study

In the Swedish study, 109 patients with ICD‐10 codes G70.0 or G70.9, who were followed at the neurological departments at the hospitals in Jönköping, Eksjö, or Värnamo during the years 2010–2014, were identified. Nineteen patients were dead, four had moved out of the region, and 16 patients did not have a MG diagnosis. A total of 70 patients with confirmed MG diagnosis were identified from the medical records, out of which 40 patients agreed to take part in the study (response rate 57.1%). The responders and nonresponders did not differ from each other (20 men and 20 women with their mean age 64.3 ± 16.2 years in responder's group; 16 men and 14 women among nonresponders with mean age 67.3 ± 16.5 years, *p* > .05).

### 2.3 Questionnaires, clinical, and neurophysiological evaluation

All identified patients were given two questionnaires (during the clinical visit or at home); (1) self‐administered questionnaire for MG patients (Maniaol, Brunborg, & Tallaksen, 2010), including questions about demographic data, MG disease‐specific data, other diseases and lifestyle issues; and (2) mental fatigue assessment; Fatigue Severity Scale (FSS) (Valko, Bassetti, Bloch, Held, & Baumann, 2008). FSS is a questionnaire with nine questions estimating the fatigue severity in different situations during the past week. Grading ranges from 1 (strong disagreement) to 7 (strong agreement) where the final score is the mean value of the nine items, and a score ≥4 is interpreted as fatigue (Valko et al., 2008). Medical records were reviewed for history of thymectomy, thymus histology, and antibody status. Current MG status of the patients was evaluated clinically by EW and LS (both board‐certified neurologists) by the Quantitative Myasthenia Gravis Score (QMG). The QMG score consists of 13 items with a global score ranging from 0 to 39; which is obtained from ocular, bulbar, respiratory, and limb muscle function. Higher score on QMG expresses more severe myasthenic deficit. EW and ML performed RNS to analyze disturbed neuromuscular transmission. Low frequency, 3 Hz, RNS was performed and amplitude decrement was calculated as the difference of amplitude between the first and fourth compound motor action potentials of the anconeus, abductor pollicis brevis, trapezius, and nasalis muscles, where a decrement of ≥10% was considered abnormal. If data of antibodies were missing, serum analysis of antibodies against the AChR was done and analysis of antibodies against MuSK was performed in AChR antibody seronegative patients, with radioimmunoassay at Karolinska Institute, Stockholm, Sweden. Clinical subdivision was defined according to early‐onset MG (EOMG, <50 years of age) and late‐onset MG (LOMG, ≥50 years of age). Subtype classification also included clinical phenotype: ocular, bulbar, and generalized MG.

### 2.4 Statistical analysis

The crude and age‐ and sex‐adjusted prevalence rates (PRs) were calculated using denominators derived from Statistics Estonia and Statistics Sweden. In order to compare the two cohorts, we standardized the rates to European standard population (http://www.who.int/healthinfo/paper31.pdf). Prevalence rates were calculated per million inhabitants and 95% confidence intervals (95% CI) were calculated for rates using Poisson distribution. Student *t* test (for parametric data) or Mann–Whitney *U* test (for nonparametric data) was used for analyzing continuous variables. Chi‐square test evaluated differences in categorical data and Fisher's exact test was used if the sample was not large and expected values were less than 5. A statistical level of significance of 5% was used (*p* < .05). The statistical analysis was performed using StatsDirect version 3.0.171 (StatsDirect Ltd., Chesire, UK).

## 3 Results

### 3.1 Demographics and antibody data

The crude PR was 234.5 (95% CI 184.5–294.0) per million population in Estonia and 203.3 (95% CI 158.5–256.9) per million population in Sweden. Age‐ and sex‐adjusted PRs are shown in Table 1. The current age of the patients did not differ (60.9 ± 18.3 years in Estonia and 64.3 ± 16.2 years in Sweden, *p* = .39). Although their age at disease onset was similar, the overall female to male ratio was different (2:1 in Estonia vs. 1:1 in Sweden). In Sweden, women had an earlier disease onset than men (35.9 ± 20.3 years for women vs. 64.7 ± 11.8 years for men, *p* = .004), whereas this difference was not significant in Estonia (51.2 ± 20.7 vs. 55.9 ± 14.8, *p* = .48). In Sweden, the female to male (F:M) ratio was 8.5:1 in the subgroup of EOMG and 1:5.3 in the subgroup of LOMG. On the other hand, the F:M ratio in Estonia was 4:1 in the EOMG subgroup and merely 1.3:1 in the LOMG subgroup. The basic demographic characteristics of the patients are shown in Table 2. None of the patients were MuSK antibody seropositive (MuSK+). In Sweden 85% of patients were AChR antibody seropositive (AChR+) and in Estonia the prevalence of AChR+ MG was 72%.

**Table 1:** Age and sex‐adjusted prevalence rates of myasthenia gravis in Southern‐Estonia and Jönköping county in Sweden on 1 January 2015

	Estonia		Sweden	
	Rate per million (95% CI)	European standard rate (95% CI)	Rate per million (95% CI)	European standard rate (95% CI)
Overall MG	230.7 (178.4–283.1)	172.6 (131.1–214.1)	198.9 (152.2–245.6)	148.1 (110.8–185.4)
Sex				
Men	180.1 (113.3–246.9)	158.0 (98.8–217.1)	302.2 (202.2–402.3)	142.8 (94.0–191.7)
Women	275.4 (196.4–354.4)	190.8 (129.1–252.4)	197.9 (131.3–264.6)	161.6 (103.1–220.0)
Age, years				
0–19	0		0	
20–29	107.2 (34.8–250.1)		44.0 (5.3–159.0)	
30–39	176.2 (70.8–362.9)		153.6 (56.4–334.3)	
40–49	94.0 (25.6–240.7)		87.2 (23.8–223.3)	
50–59	259.4 (129.5–464.1)		236.6 (113.5–435.1)	
60–69	284.2 (136.3–522.7)		361.5 (202.3–596.3)	
70–79	909.1 (593.8–1,332.0)		677.0 (413.6–1,045.6)	
>80	678.2 (350.5–1,184.7)		651.8 (347.1–1,114.6)	

95% CI, 95% confidence interval.

**Table 2:** Basic demographic data of patients with myasthenia gravis in Estonia and Sweden

	Estonia *N* = 36	Sweden *N* = 40	*p*‐Value
Gender, *N* (%)			
Male	12 (33.3)	20 (50)	.22
Female	24 (66.7)	20 (50)	
Age onset, mean ± *SD*	52.8 ± 18.8	49.5 ± 22.1	.50
Civilization status, *N* (%)			
Alone	8 (22.9)	4 (10)	.23
Married/coupled	22 (62.9)	32 (80)	.16
Widow	5 (14.3)	4 (10)	.73
Education, *N* (%)			
Basic	6 (17.1)	19 (48.7)	.01^a^
Secondary	8 (22.9)	6 (15.4)	.60
Vocational	10 (28.6)	3 (7.7)	.04^a^
Higher	11 (31.4)	11 (28.2)	.96
Working status, *N* (%)			
Full‐time/part‐time worker	13 (37.1)	9 (22.5)	.26
Disability retirement	5 (14.3)	2 (5)	.24
Retired	15 (42.9)	25 (62.5)	.14
Other	2 (5.7)	4 (10)	.68

Results are presented as number (%) and mean ± *SD*. *SD*, standard deviation.

a Significant difference (*p* < .05).

### 3.2 Physical versus mental fatigue

The initial MG symptoms reported by the patients did not differ (Table 3) and the proportion of clinical subgroups (ocular, bulbar, generalized) was comparable (Table 4). There was a tendency for Swedish patients to report no myasthenic symptoms within the last 3 months (9 [23.1%] vs. 3 [8.6%], *p* = .09). Abnormal decrement was detected in a higher proportion (83.9%) of Estonian patients compared to Swedish patients (55.9%; *p* = .02).

**Table 3:** Symptoms at onset and during the three last months in patients with myasthenia gravis in Estonia and Sweden

Symptoms, *N* (%)	At disease onset		*p*‐Value	Currently		*p*‐Value
	Estonia	Sweden		Estonia	Sweden	
Ptosis	20 (58.8)	26 (72.2)	.24	18 (52.9)	12 (31.6)	.07
Diplopia	22 (64.7)	24 (61.5)	.78	17 (50.1)	12 (30.8)	.10
Weakness of upper limb muscles	20 (58.8)	22 (56.4)	.84	21 (61.8)	15 (38.5)	<.05^a^
Weakness of lower limb muscles	19 (55.9)	18 (46.2)	.41	19 (55.9)	16 (41)	.21
Weakness of neck muscles	15 (44.1)	19 (48.7)	.70	12 (35.3)	14 (35.9)	.10
Weakness of face muscles	13 (38.2)	18 (47.4)	.44	10 (29.4)	14 (36.8)	.51
Dysarthria	18 (52.9)	19 (48.7)	.72	12 (35.3)	9 (23.1)	.25
Chewing difficulties	18 (52.9)	17 (43.6)	.43	11 (32.4)	4 (10.3)	.02^a^
Dysphagia	12 (36.4)	17 (43.1)	.54	10 (29.4)	6 (15.4)	.15
Dyspnea	13 (38.2)	21 (53.9)	.19	16 (47.1)	13 (33.3)	.24

Results are presented as number (%).

a Significant difference (*p* < .05).

**Table 4:** Clinical characteristics on myasthenia gravis patients in Estonia and Sweden

	Estonia	Sweden	*p*‐Value
Subgroups, *N* (%)			
Juvenile MG	0	2 (5.3)	.49
Early‐onset MG	15 (42.9)	16 (44.4)	>.99
Late‐onset MG	20 (57.1)	20 (55.6)	.88
Phenotypes, *N* (%)			
Generalized	29 (80.6)	31 (79.5)	>.99
Bulbar	3 (8.3)	2 (5.1)	.67
Pure ocular	4 (11.1)	6 (15.4)	.74
Antibody serology, *N* (%)			
AChR ab	23 (71.9)	34 (85)	.06
MuSK ab	0	0	
SNMG	9 (28.1)	6 (15)	.42
Missing antibodies	4 (11.1)	0	<.05*
Thymectomy, *N* (%)	14 (38.9)	19 (47.5)	.60
Thymoma MG, *N* (%)	2 (5.5)	4 (10.0)	.77

MG, myasthenia gravis; AChR ab, acetylcholine receptor antibodies; MuSK ab, muscle‐specific kinase antibodies; SNMG, seronegative myasthenia gravis. Results are presented as number (%). *Significant difference (*p* < .05).

At disease onset, symptoms were moderately expressed in both countries (in 10‐point scale, where 1 means almost no symptoms and 10 means very pronounced symptoms) (6.6 ± 2.9 in Estonia vs. 6.6 ± 2.4 in Sweden, *p* = .99). Estonian patients judged their current health as poor (5.6 ± 2.8), compared to the Swedish patients (3.4 ± 2.3, *p* = .0005). Albeit, the patients' objective MG fatigue (QMG) did not differ (5.0 ± 3.7 in Estonia and 5.4 ± 4.4 in Sweden, *p* = .68); the patients' self‐perceived health positively correlated with the QMG score (*R* = .56, *p* < .0001). Further, a strong positive correlation was found between self‐related health and FSS (*R* = .52, *p* < .0001) and the mean FSS was considerably higher in Estonia (5.0 ± 1.7) compared to Sweden (3.5 ± 1.6; *p* = .001). The most commonly mentioned factor affecting health status was psychological stress (54.6% in Estonia and 43.6% in Sweden) and infection (59.1% in Estonia and 35.9% in Sweden) in both groups followed by physical stress (50% in Estonia and 35.9% in Sweden; *p* > .05 for all). Estonian patients more frequently reported medications as a cause of health status deterioration (36.4% vs. 10.3%, *p* = .01) and Swedish patients marked “other” as a deteriorative factor (not infection, psychological or physical stress, or weather; 13.6% in Estonia and 41% in Sweden, *p* = .03).

### 3.3 Physical activity and treatment regimens

A significant difference was found in levels of physical activity, where Swedish patients stated higher daily physical activity levels. While only one Swedish patient reported no physical activity (e.g., walking, cycling, gardening) at all, the percentage of physical inactivity was 38.2% among Estonian responders (*p* = .004). A similar situation was found regarding regular physical exercise; 71.9% of Estonian patients did not perform physical exercise training at all compared to 25.8% in Sweden (*p* = .004).

The number of patients on >1 treatment regimen was similar in the two countries (36.1% in Estonia and 27.5% in Sweden, *p* = .42). Although fewer patients in Estonia were treated with prednisone, the difference was not significant (22.9% vs. 38.5%, *p* = .15). More patients were currently on azathioprine treatment in Estonia (*p* = .04) and the daily doses of pyridostigmine were also higher in this cohort (367 ± 199 mg in Estonia vs. 231 ± 161 mg in Sweden, *p* = .007). A comparable number of patients had been thymectomized and a thymoma was detected in a minor fraction of patients in both countries. The spectrum of concomitant diseases revealed that the frequency of autoimmune thyroiditis was considerably higher among the Estonian patients (28.6% vs. 8.8%, *p* = .04).

## 4 Discussion

This is the first study employing questionnaires, objective clinical scoring, and neurophysiological reexamination of MG patients from comparable counties in two different European countries. The data indicate that perceived health satisfaction was significantly lower among Estonian than Swedish patients, although objective MG status was comparable. Albeit, similar procedures exist for diagnosis and treatment of MG, it is intriguing that Estonian patients perceived their health situation as worse. Since the objective clinical measure QMG did not differ, we cannot exclude that the reasons for this could be socioeconomical and/or psychological in nature. Self‐rated health has been the poorest in the former Soviet Union and a strong correlation with economic status exists (Carlson, 1998, 2004). In addition, welfare measures such as social benefits and services are important for population health, where Scandinavian welfare regimes have advantages compared to Eastern European systems (Olsen & Dahl, 2007). Two former studies compared Estonian and Scandinavian (Norwegian) patients with post‐polio and spinal cord injury (Rekand et al., 2003; Sabre et al., 2013). Both studies concluded that fewer Estonian patients were working full‐time and more Estonian patients were unemployed and reported low income (Rekand et al., 2004; Sabre et al., 2013). In line with these previous studies, our study confirms that a high percentage of Estonian patients with MG were physically inactive. Nevertheless, we found no differences in working status among MG patients in Estonia and Sweden. Further, neither marital status nor education was correlated with perception of poor wellbeing. Although the general education level was slightly higher in the Estonian cohort, the percentage of individuals with higher education was the same in both countries.

Fatigue consists of both physiological and psychological aspects (Elsais, Wyller, Loge, & Kerty, 2013) and relates to cognitive performance as well as functional ability (Elsais et al., 2013; Valko et al., 2008). In MG, physical muscle fatigue is typically considered the most important contributory factor. Nevertheless, even during stable pharmacological remission without objective muscle fatigue, MG patients reported higher levels of mental fatigue compared to healthy controls (Elsais et al., 2013). Although patients with chronic diseases often experience anxiety and depression (Suzuki et al., 2011), this does not explain why MG patients in Estonia experienced higher mental fatigue compared to MG patients in Sweden. One possible reason for differences is that the general population in Estonia has a high prevalence of depressive symptoms, which is in part connected to economic factors (Aluoja, Leinsalu, Shlik, Vasar, & Luuk, 2004) and in turn results in lower self‐related health scores. Although we did not include specific measurements of depression or anxiety in this study, the results from the FSS form demonstrate that mental fatigue severity influenced the subjective symptom evaluation.

As MG is a disease with exacerbations and remissions, obtaining a stable disease course already in the very beginning of the treatment is a challenge (Suzuki et al., 2011). There are treatment guidelines; however, most treatment strategies are based on uncontrolled observational studies, case series, or good clinical practice (Kerty, Elsais, Argov, Evoli, & Gilhus, 2014; Skeie et al., 2010) and hence, patients may receive suboptimal treatment. Although, the immunosuppressive treatment was not different in the two regions, Estonian patients were prescribed higher doses of acetylcholinesterase inhibitors (AChEI) than Swedish patients. Importantly, mental fatigue could be misinterpreted as physical weakness and result in increased doses of AChEI from the treating physician, which paradoxically could increase symptomatic fatigue even more. AChEIs are also known to alter autonomic function that is related to fatigue, and therefore patients using them may report more severe mental fatigue. Especially, older patients are known to be more prone to develop cholinergic side effects from AChEI (Punga, Sawada, & Stalberg, 2008).

The PRs of MG were similar in Estonia and Sweden, having increased in both regions during the last decade (Kalb, Matell, Pirskanen, & Lambe, 2002; Oopik, Kaasik, & Jakobsen, 2003). We agree with the conclusion of the previous study (Oopik, Puksa, Luus, Kaasik, & Jakobsen, 2008) that MG might be overdiagnosed in Estonia. Although inclusion criteria were the same, prevalence and gender ratios differed between the countries which could possibly have affected the results. The reason for those differences is beyond the scope of this study, but would be necessary to look further into.

A novel north‐south gradient has been described for MuSK+ MG with higher prevalence in southern European countries (Boldingh et al., 2015). Although, MuSK antibody analysis is not yet routine in clinical practice in Estonia and no information was found concerning MuSK antibody status in the medical records, on reexamination of Estonian patient samples, we found no MuSK+ patients, as expected from this Northern latitude.

The main strengths of the study were that practically all cases in the two regions were found, the regions were eligible, and that subjective evaluations from the patients were compared to objective clinical observations. One limit of the study is the possibility of selection bias. A relatively small number of participants preclude wide‐ranging conclusions. The response rate was quite similar in the two countries and the responders did not differ from nonresponders by gender or age. Secondly, as we found overdiagnosis of MG in Estonia, the calculated PRs must be interpreted with a certain amount of caution.

In conclusion, although MG patients in the two selected European regions objectively had the same muscle fatigue status, Estonian patients reported a worse subjective health state with more severe mental fatigue than Swedish patients. These possible regional differences are important to keep in mind when interpreting data from international trials.

## Conflict of Interest

The authors declare no conflict of interest.
